# HOUSE CALLS AND BEHAVIORAL HEALTH INTEGRATION: ADDRESSING MENTAL HEALTH LITERACY AND DEPRESSION CARE

**DOI:** 10.1093/geroni/igz038.503

**Published:** 2019-11-08

**Authors:** Elyse Perweiler, Lisa Bodenheimer, Gail Belfer, Jennifer DeGennaro, Anita Chopra, Marla Meyers

**Affiliations:** 1 Rowan School of Osteopathic Medicine, Stratford, New Jersey, United States; 2 Rowan University School of Osteopathic Medicine, Stratford, New Jersey, United States; 3 Jewish Family & Children’s Service of Southern New Jersey, Cherry Hill, New Jersey, United States

## Abstract

The House Calls and Behavioral Health Integration project was an inter-agency collaboration to address depression in the older adult population by increasing knowledge and awareness of the condition and providing better access to care. Rowan Medicine, New Jersey Institute for Successful Aging (NJISA) and Jewish Family and Children Services (JFCS) partnered to deliver a comprehensive education, screening and referral program for residents of 3 senior housing facilities serving an estimated 300 elderly and disadvantaged residents. The goal of this year long project was to provide depression education for both housing residents and staff; offer on-going access to free depression screens (using PHQ-9) for residents; and refer residents to an appropriate resource (counseling/case management/house calls) depending on depression screen outcome. Depression education was provided to 15 housing staff and 78 older adult residents. A total of 34 depression screens were completed (82% female, average age=82 years); further evaluation of symptoms was indicated for 59% (n=20) of those screened. Seventeen of the 20 residents accepted a recommendation for a referral to supportive counseling; 3 declined. The average PHQ-9 score was 8.45 (SD=4.42; Range 1-24). Referrals to case management and house calls (n=4) were also made. Feedback from both housing residents and staff was positive and the project team learned valuable lessons about serving older adults in congregate living settings which has informed other programming. The project was successful in providing education, screening and referrals to residents who might not otherwise have access to specialized geriatric behavioral health interventions.

